# Supported l-tryptophan on Fe_3_O_4_@SiO_2_ as an efficient and magnetically separable catalyst for one-pot construction of spiro[indene-2,2′-naphthalene]-4′-carbonitrile derivatives†

**DOI:** 10.1039/d1ra07654j

**Published:** 2022-01-05

**Authors:** Aref Ghasemi-Ghahsareh, Javad Safaei-Ghomi, Hourieh Sadat Oboudatian

**Affiliations:** Department of Organic Chemistry, Faculty of Chemistry, University of Kashan P. O. Box 87317-51167 Kashan I. R. Iran safaei@kashanu.ac.ir +98-31-55552935 +98-31-55912385

## Abstract

In this work, l-tryptophan functionalized silica-coated magnetic nanoparticles were readily prepared and evaluated as a recyclable magnetic nanocatalyst for the synthesis of spiro[indene-2,2′-naphthalene]-4′-carbonitrile derivatives through the one-pot four-component reaction of malononitrile, cyclohexanone, aromatic aldehydes, and 1,3-indandione. This novel magnetic nanocatalyst was confirmed to be effective and provide products in moderate to excellent yields under reflux conditions. The structure of obtained nanoparticles was characterized using FT-IR, XRD, VSM, EDX, elemental mapping, FE-SEM, and TGA. This synthetic protocol provides several benefits such as excellent yields in short reaction times (64–91%), saving costs, reusability of the catalyst using an external magnet (seven runs), and low catalyst loading.

## Abstract 2

l-Tryptophan functionalized silica-coated magnetic nanoparticles were prepared and evaluated as a magnetic nanocatalyst for the synthesis of spiro[indene-2,2′-naphthalene]-4′-carbonitrile derivatives through the one-pot four-component reaction.

## Graphical abstract

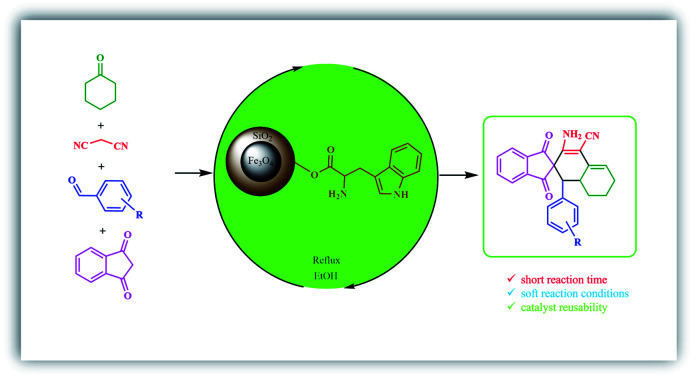

## 1. Introduction

Multicomponent reactions (MCRs) and associated one-pot synthesis such as domino, tandem and cascade reactions have always been one of the most significant scopes in the development of new methodologies in organic synthesis and catalysis due to their great applications, particularly in pharmaceutical studies.^1–4^ MCRs often produce highly complex molecules from simple precursors, thus, avoiding complicated purification steps and saving solvents, reagents and times.^5–7^

**Scheme 1:**
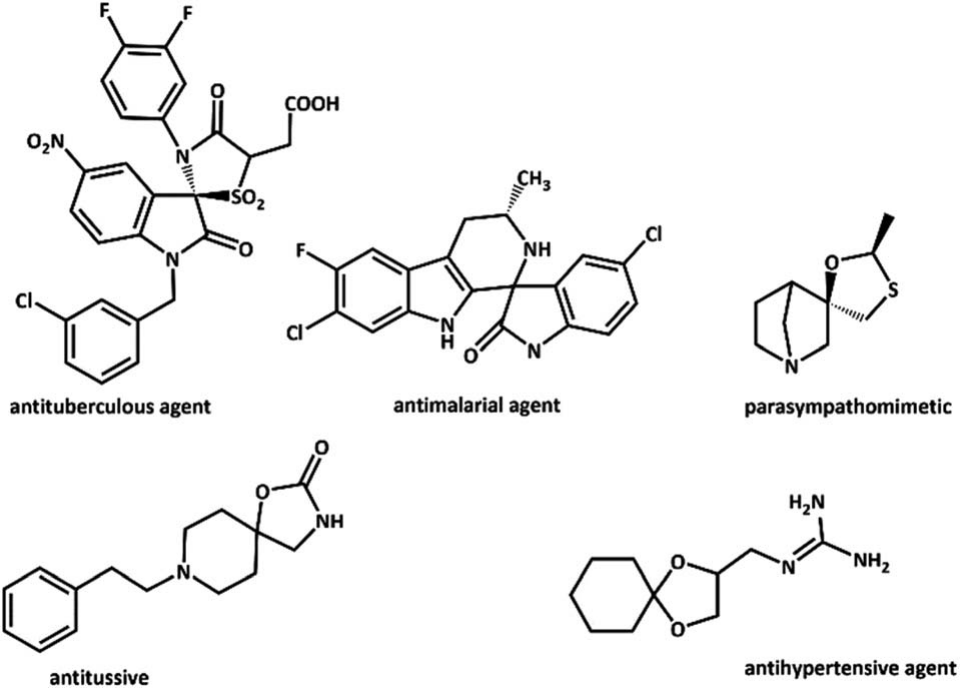
Relevant bioactive compounds containing spiro frameworks.

Spiro compounds show a broad range of effective performance in pharmacology. They have a large number of pharmacological and biological properties such as anti-cancer,^8^ antimicrobial,^9^ anti-diabetic,^10^ antitumor,^11^ and anti-hypertensive^12^ activities due to their fascinating structures (Scheme 1). Among the spiro family members, spirocarbocycles are imperative scaffolds known for their extensive pharmacological significance that are found in numerous products and bioactive molecules. It is worth pointing out that, MCRs are one of the most potent techniques for the production of spiro compounds.^13,14^ Furthermore, vinylogous Michael addition was reported as a key step in the preparation of many spiro compounds.^15–17^

In recent years, a few synthesis methods for the synthesis of spiro[indene-2,2′-naphthalene]-4′-carbonitriles have been reported in the literature. l-Proline,^18^ ethylene glycol^19^ and quaternary ammonium surfactant [C_18_-Dabco][Br]^20^ have been utilized in these methods. Nevertheless, there are drawbacks with these reported strategies, for instance, prolonged reaction time, catalyst toxicity, and more importantly the catalyst reusability is difficult. However, further studies are still essential for the efficient, environmental and economical multicomponent methodology for the synthesis of these spiro compounds, even though each of the known methods for the synthesis of spiro[indene-2,2′-naphthalene]-4′-carbonitrile compounds has its competency.

In general, magnetic nanoparticles have been applied for a range of biomedical applications, particularly in the areas of medical imaging, diagnostics, and treatment.^21–25^ Additionally, they have been commonly employed in many organic reactions because of their easy removal and convenient separation using an external magnetic field.^26–29^ Among all magnetic nanoparticle types, Fe_3_O_4_ nanoparticles (NPs) have received more attention owing to their exclusive features such as superparamagnetic nature, non-toxic, biocompatibility, and facile synthesis process from accessible precursors (*e.g.*, ferrous and ferric salts).^30^ Also, Fe_3_O_4_ nanoparticles possess plenty of hydroxyl groups (OH) on their surfaces, thus, they are normally hydrophilic. However, the naked Fe_3_O_4_ is not stable since it can be easily oxidized into the other substances in the air, therefore, coating materials or modification is critical in order to prevent them from oxidation. For this purpose, silica (SiO_2_) has been widely considered as one of the best protection shells since it is inexpensive, has a high specific surface area, and has high resistance under catalytic conditions. It is noteworthy that the functional groups containing oxygen, which are distributed on the surface of Fe_3_O_4_@SiO_2_ can act as active sites.

l-Tryptophan is known as a chiral molecule. Environmental-friendliness, less-expensive, and easy availability are some of the benefits of l-tryptophan. Moreover, this α-amino acid can participate in many organic reactions as a catalyst because it possesses active site groups (amino group and carboxylic group). In other words, l-tryptophan can be broadly used in base-catalyzed organic reactions.^31^ However, tough recovery and prolonged reaction times are prominent drawbacks of this catalyst. Several strategies can be used to overcome the above-mentioned problems such as co-catalysts, varying types of supports such as ionic liquids,^32^ polymers,^33^ and silica.^34^

In this study, we introduce a straightforward approach for the fabrication of Fe_3_O_4_@SiO_2_-l-tryptophan as a robust inorganic-organic hybrid catalyst. This heterogeneous, environmentally benign, and highly reusable catalyst represents high catalytic activity to synthesize spiro[indene-2,2′-naphthalene]-4′-carbonitrile derivatives *via* a one-pot, four-component condensation reaction of malononitrile (1 mmol), cyclohexanone (1 mmol), various aromatic aldehydes (1 mmol) and 1,3-indandione (1 mmol) under reflux conditions in EtOH.

## 2. Result and discussion

### 2.1 Preparation and characterization of Fe_3_O_4_@SiO_2_-l-tryptophan catalyst

**Scheme 2:**
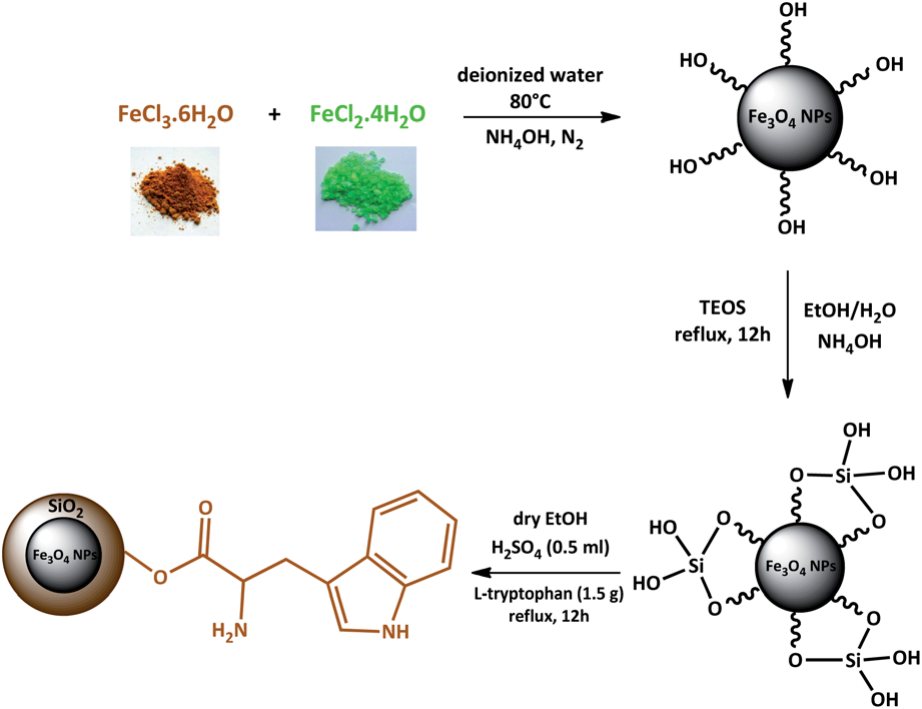
Schematic for the preparation of Fe_3_O_4_@SiO_2_-l-tryptophan nanocatalyst.

Magnetic nanoparticles (MNPs) were prepared using Fe^2+^ and Fe^3+^ ions by the co-precipitation method. Then, Fe_3_O_4_ NPs coated by the silica network provided reaction sites for further functionalization and thermal stability. In the end, Fe_3_O_4_@SiO_2_ was reacted with l-tryptophan in the presence of H_2_SO_4_ in order to produce Fe_3_O_4_@SiO_2_-l-tryptophan nanoparticles (Scheme 2). Of note, the l-tryptophan layer is linked on the surface of Fe_3_O_4_@SiO_2_ by chemical adsorption.^35^

**Fig. 1:**
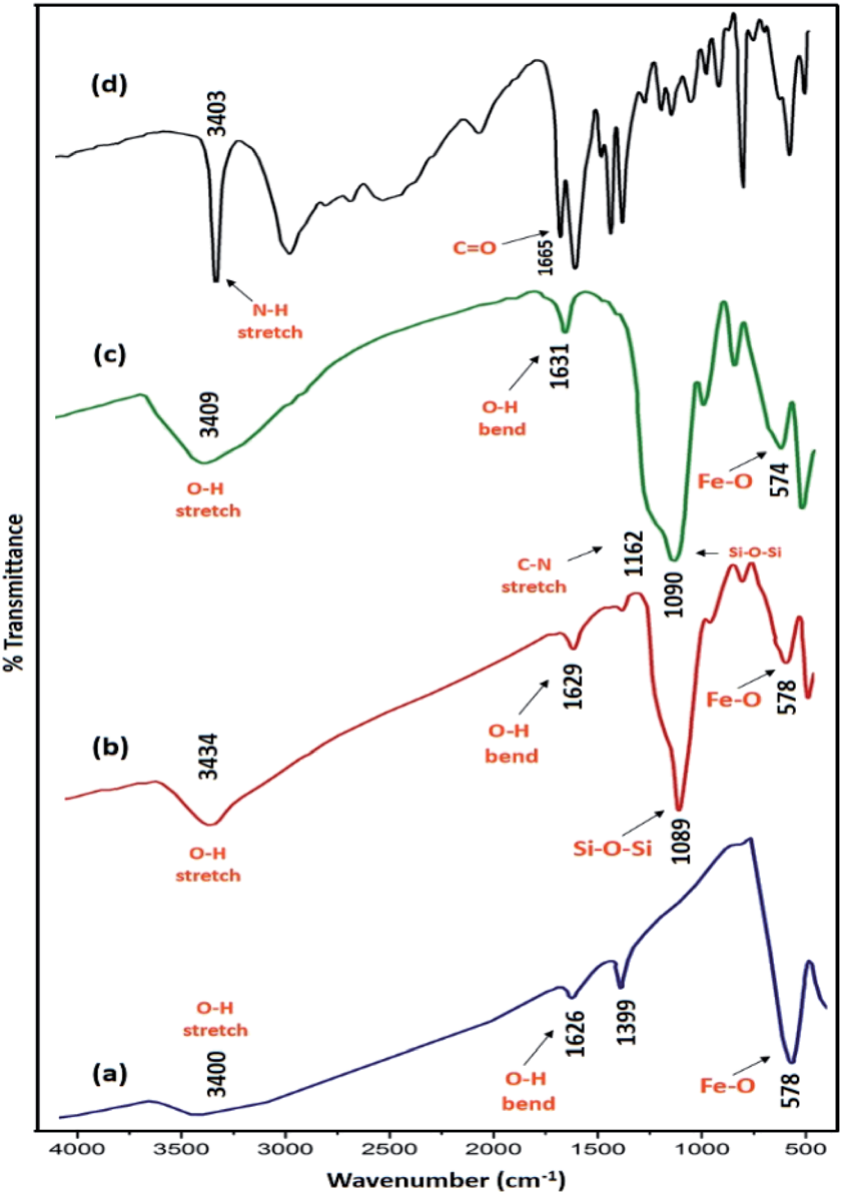
FT-IR spectra of (a) Fe_3_O_4_, (b) Fe_3_O_4_@SiO_2_, (c) Fe_3_O_4_@SiO_2_@l-tryptophan and (d) l-tryptophan.

Fig. 1 illustrates FT-IR spectra of Fe_3_O_4_, Fe_3_O_4_@SiO_2_, Fe_3_O_4_@SiO_2_-l-tryptophan, and l-tryptophan. It can be observed that the band in the region of 578 cm^−1^ is attributed to vibrations of the Fe–O bond and bands at 3400 and 1626 cm^−1^ are ascribed to O–H stretching and bending vibrations, respectively (Fig. 1a). In the Fe_3_O_4_@SiO_2_ spectrum, the strong peak at 1089 cm^−1^ could be assigned to asymmetric stretching vibrations of Si–O–Si bonds. This peak verifies that the silica layer was coated on the surface of Fe_3_O_4_ very well (Fig. 1b). For the l-tryptophan functional group, the absorption band at 1162 cm^−1^ belongs to the C–N stretching vibration and the peak at 3409 cm^−1^ corresponds to O–H stretching vibrations (Fig. 1c).

**Fig. 2:**
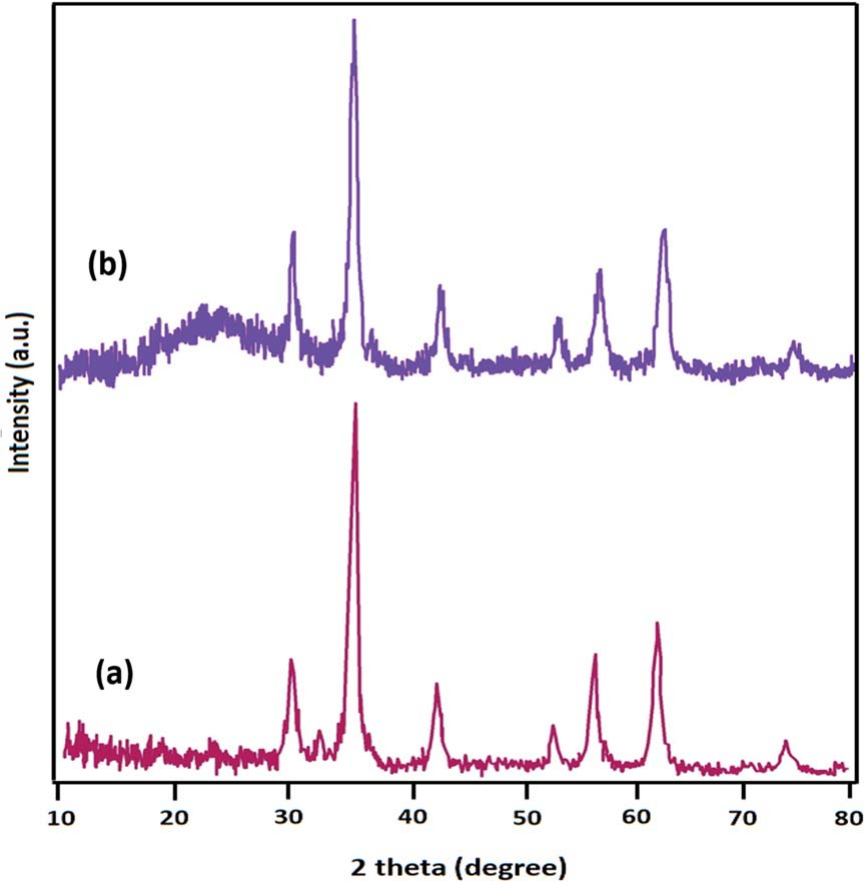
XRD patterns of (a) Fe_3_O_4_, and (b) Fe_3_O_4_@SiO_2_-l-tryptophan.

The powder X-ray diffraction (XRD) patterns of Fe_3_O_4_ and Fe_3_O_4_@SiO_2_-l-tryptophan are displayed in Fig. 2. XRD data clearly demonstrated 6 diffraction angles (2*θ*) at 30.2°, 35.5°, 43.2°, 53.6°, 57.1°, and 62.8°, following standard Fe_3_O_4_ XRD patterns (JCPDS No. 75-0449) (Fig. 2a). From Fig. 2b, it can be clearly observed at about 2*θ* = 24°, which is assigned to the amorphous organic group. The diameter (*D*) of the Fe_3_O_4_@SiO_2_-l-tryptophan NPs was calculated using the Scherrer equation (*D* = *Kλ*/(*β* cos *θ*)), where *θ* is the Bragg angle of the maximum of the diffraction peak and *β* is the line broadening at half the maximum, while *λ* is the X-ray wavelength (0.154 nm for CuKα). *K* is a dimensionless shape factor, which usually takes a value of about 0.9.

**Fig. 3:**
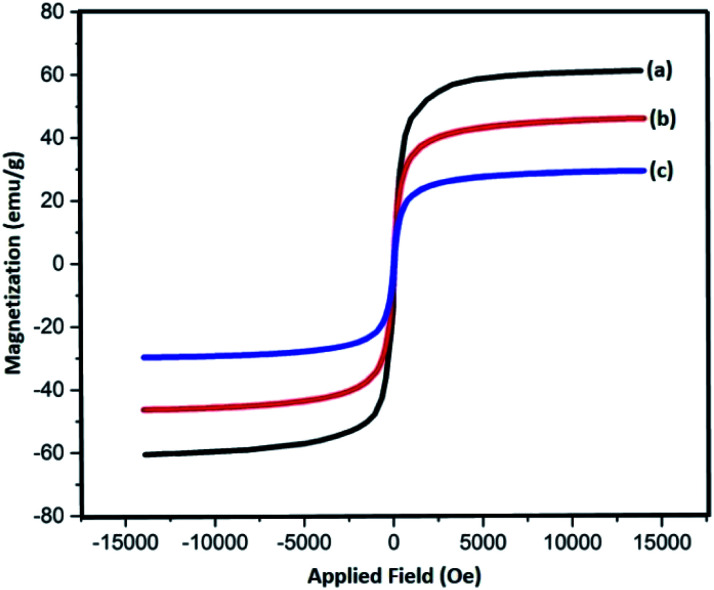
VSM curves of (a) Fe_3_O_4_, (b) Fe_3_O_4_@SiO_2_ and, (c) Fe_3_O_4_@SiO_2_-l-tryptophan nanoparticles.

Magnetic properties of Fe_3_O_4_, Fe_3_O_4_@SiO_2_, and Fe_3_O_4_@SiO_2_-l-tryptophan nanoparticles were measured with the help of a vibrating sample magnetometer (VSM) (Fig. 3). According to these results, all three samples are superparamagnetic at 60.7 emu g^−1^, which belongs to Fe_3_O_4_ NPs showing the highest value of saturation magnetization (Ms). Additionally, the magnitude of saturation magnetization values for Fe_3_O_4_@SiO_2_ and Fe_3_O_4_@SiO_2_-l-tryptophan are 48.23 emu g^−1^ and 29.64 emu g^−1^, respectively. These results illustrate that the magnetization property decreases after coating and functionalization.

**Fig. 4:**
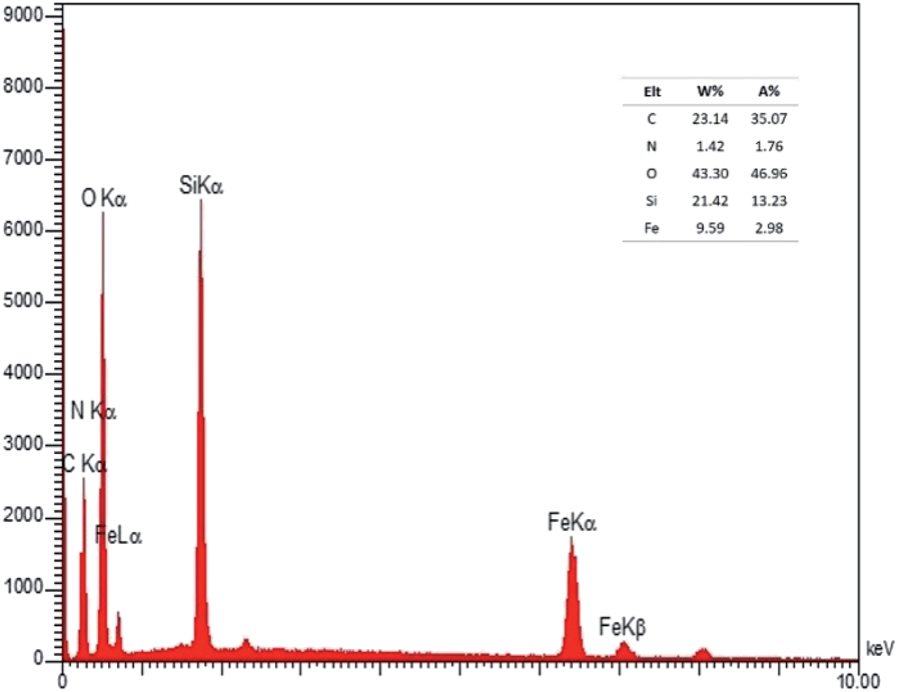
EDX analysis of Fe_3_O_4_@SiO_2_-l-tryptophan nanoparticles.

**Fig. 5:**
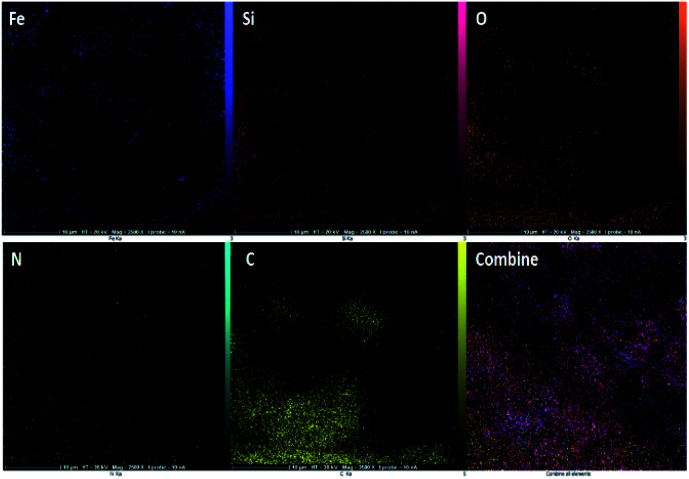
Elemental mapping images of Fe_3_O_4_@SiO_2_-l-tryptophan nanoparticles.

The presenting elements can be clearly seen in the structure of Fe_3_O_4_@SiO_2_-l-tryptophan using energy-dispersive X-ray spectroscopy (EDX) (Fig. 4). Besides, all elements are well distributed throughout the Fe_3_O_4_@SiO_2_-l-tryptophan, which is revealed by elemental mapping images (Fig. 5).

**Fig. 6:**
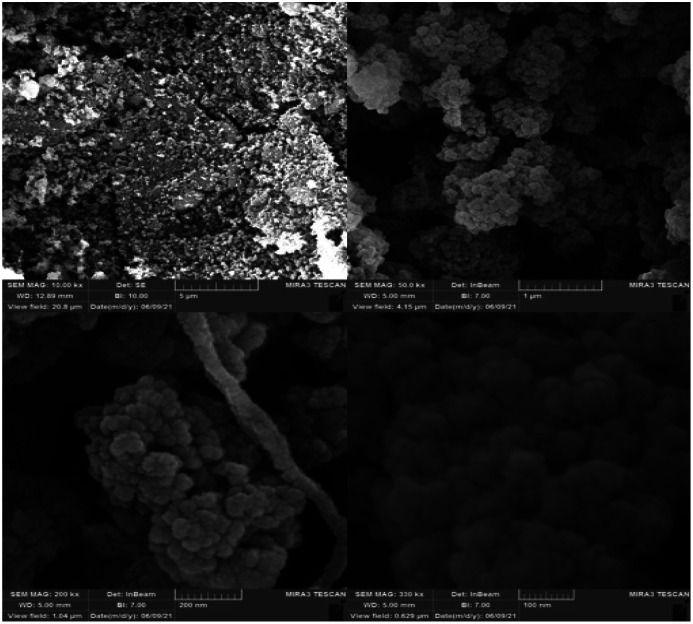
FE-SEM photographs of Fe_3_O_4_@SiO_2_-l-tryptophan nanoparticles.

In order to study the morphology, uniformity and size of the catalyst, FE-SEM was used. As it is obvious from Fig. 6, SEM images of the sample indicate that the prepared nanoparticles have a uniform size, spherical shape, and disordered mesosphere. The average size of Fe_3_O_4_@SiO_2_-l-tryptophan MNPs was calculated to be about 37 nm.

**Fig. 7:**
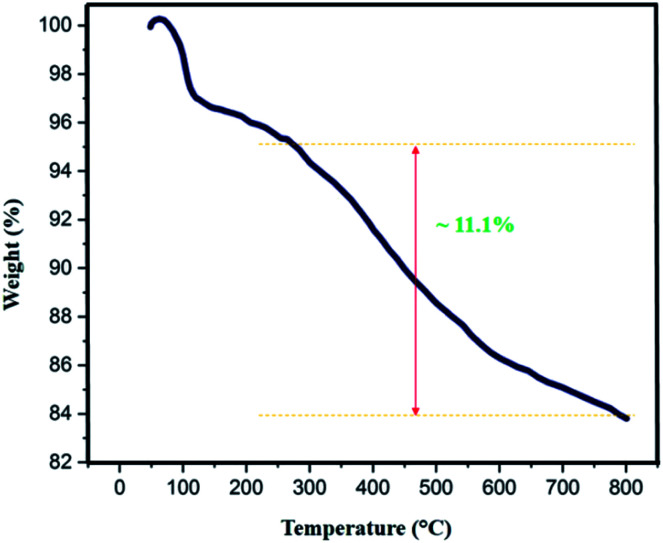
TGA curve of Fe_3_O_4_@SiO_2_-l-tryptophan NPs.

The thermal behavior of Fe_3_O_4_@SiO_2_-l-tryptophan nanoparticles was estimated by TGA analysis (Fig. 7). Results depict appropriate thermal stability with no significant decline in the weight. The weight loss at low temperatures (<∼100 °C) can be either due to the removal of surface –OH groups or physically adsorbed solvent molecules trapped in the SiO_2_ layer. According to the curve, the observed weight loss of about 11.1 (%) above 250 °C can be because of the decomposition of l-tryptophan group, which is attached to the silica layer. Therefore, the TGA curve reveals that Fe_3_O_4_@SiO_2_-l-tryptophan NPs is stable up to 250 °C and is certainly fit for the synthesis of spiro[indene-2,2′-naphthalene]-4′-carbonitrile compounds.

In order to calculate the extent of the functionalization per SiO_2_ group, weight loss values were employed together with the molecular weight of diverse moieties, and eqn (1) was used for the calculation.^36^ In this equation, *X* stands for the number of sample SiO_2_ groups per each covalent functional group (l-tryptophan), *R* (%) is the residual mass at 800 °C in the TGA plot, *L* (%) is the weight loss in the range of 100–800 °C, and *M*_w_ is the molecular weight of the desorbed functional groups. Taking into account that the covalently l-tryptophan measurements depicted one functionality every ∼25 SiO_2_ groups in the Fe_3_O_4_@SiO_2_-l-tryptophan.1
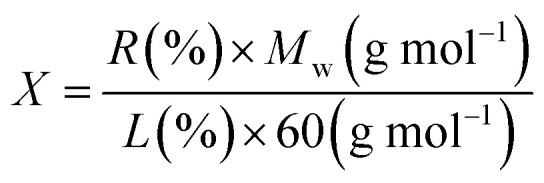


### 2.2 Comparison of the efficiency of solvents, amount of Fe_3_O_4_@SiO_2_-l-tryptophan NPs, and other catalysts on the synthesis of spiro[indene-2,2′-naphthalene]-4′-carbonitriles

The tandem one-pot reaction of malononitrile, cyclohexanone, 4-nitro benzaldehyde, and 1,3-indandione was chiefly selected as a model reaction in order to observe the catalytic activity of the prepared Fe_3_O_4_@SiO_2_-l-tryptophan. To optimize the reaction, we utilized different conditions. At first, the effect of the various electron-pair donor (EPD) and electron-pair acceptor (EPA) solvents were studied to assess the best reaction conditions. According to the results displayed in Table 1, EPD and protic solvents (acting as Lewis bases) showed better performance in the reaction. Based on the reaction mechanism, protic solvents surrounded carboanion and carbocations, hence, the activation energy was decreased.^37^ Satisfactory result were observed in terms of the yield and reaction time in the EtOH solvent. Besides, when the reaction was carried out using Fe_3_O_4_@SiO_2_-l-tryptophan NPs as the catalyst in EtOH, the product was obtained in excellent yields in a very short time (Table 1, entry 7).

**Table 1:** Optimization of reaction conditions for the formation of spiro[indene-2,2′-naphthalene]-4′-carbonitriles^a^

Entry	Solvent	Catalyst	Time (min)	Yield^b^ (%)
1	H_2_O	Nano Fe_3_O_4_@SiO_2_@l-tryp (20 mg)	180	45
2	EtOH/H_2_O	Nano Fe_3_O_4_@SiO_2_@l-tryp (20 mg)	150	64
3	DMF	Nano Fe_3_O_4_@SiO_2_@l-tryp (20 mg)	180	41
4	THF	Nano Fe_3_O_4_@SiO_2_@l-tryp (20 mg)	180	—
5	CH_3_CN	Nano Fe_3_O_4_@SiO_2_@l-tryp (20 mg)	180	39
6	MeOH	Nano Fe_3_O_4_@SiO_2_@l-tryp (20 mg)	50	82
7	**EtOH**	**Nano Fe**_**3**_ **O**_**4**_ **@SiO**_**2**_ **@** **l** **-tryp (20 mg)**	**30**	**91**
8	EtOH	Morpholine	50	78
9	EtOH	TEA	180	30
10	EtOH	PTSA	180	—

a Reaction conditions: malononitrile (1 mmol), cyclohexanone (1 mmol) *p*-nitro benzaldehyde (1 mmol), and 1,3-indandione (1 mmol) in the presence of Fe_3_O_4_@SiO_2_-l-tryptophan under reflux conditions.

b Isolated yields.

As a remarkable point, when we utilized a strong base catalyst, the obtained product had a low yield (Table 1, entry 9). In addition, in the presence of the acid catalyst, no product was observed in the period of 180 min (Table 1, entry 10). According to the data, conducting the model reaction without any catalyst gave only 21% after 180 min (Table 2, entry 1). We also investigated the catalytic activity of Fe_3_O_4_@SiO_2_, l-tryptophan and Fe_3_O_4_@SiO_2_-l-tryptophan. Based on our empirical experiments, l-tryptophan, which was modified on the surface of Fe_3_O_4_@SiO_2_ indicated the best catalytic performance in the reaction. Moreover, it was discovered that in the presence of Fe_3_O_4_@SiO_2_-l-tryptophan at 20 mg, the reaction rate and yield were considerably increased (Table 2, entry 5).

**Table 2:** Reported catalytic systems for the formation of spiro[indene-2,2′-naphthalene]-4′-carbonitriles^a^

Entry	Catalyst	Time (min)	Temp. (°C)	Yield^b^ (%)	Ref.
1	—	180	Reflux	21	This work
2	Fe_3_O_4_@SiO_2_ (20 mg)	300	Reflux	42	This work
3	l-Tryptophan (20 mg)	300	Reflux	68	This work
4	Nano Fe_3_O_4_@SiO_2_@l-tryp (20 mg)	80	rt	90	This work
5	**Nano Fe**_**3**_ **O**_**4**_ **@SiO**_**2**_ **@** **l** **-tryp (20 mg)**	**30**	**Reflux**	**91**	**This work**
6	Nano Fe_3_O_4_@SiO_2_@l-tryp (15 mg)	80	Reflux	90	This work
7	Nano Fe_3_O_4_@SiO_2_@l-tryp (30 mg)	30	Reflux	91	This work
8	l-Proline (15 mol%)	180	rt	89	18
9	Ethylene glycol (3.3 g)	150	100	84	19
10	DABCO (10 mol%)	90	rt	88	20

a Reaction conditions: malononitrile (1 mmol), cyclohexanone (1 mmol) *p*-nitro benzaldehyde (1 mmol), and 1,3-indandione (1 mmol) in the presence of Fe_3_O_4_@SiO_2_-l-tryptophan in EtOH under reflux conditions.

b Isolated yields.

To investigate the scope of this protocol, various aromatic aldehydes (4), possessing both electron-donating and withdrawing groups were reacted with 1,3-indandione, malononitrile, and cyclohexanone (Table 3). The results were ascertained that aromatic aldehydes with electron-withdrawing groups reacted faster compared to those with electron-donating groups. Furthermore, the electron-withdrawing groups at the *para* position of benzaldehyde (5a, 5d, and 5j) resulted in excellent yields (Table 3).

**Table 3:** Fe_3_O_4_@SiO_2_@l-tryptophan NPs catalyzed synthesis of spiro[indene-2,2′-naphthalene]-4′-carbonitrile derivatives^a^

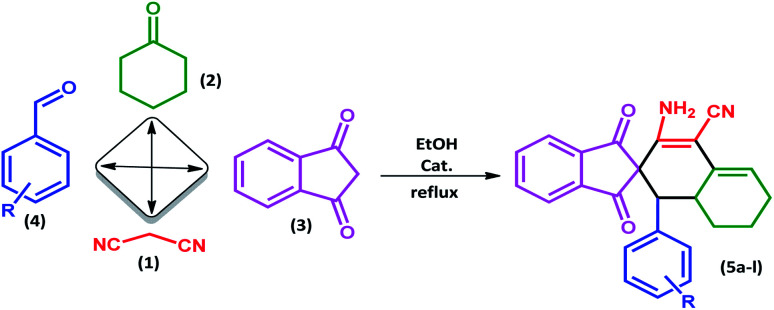					
Entry	Name	Product	Time (min)	Yield^b^ (%)	MP (°C)
1	5a	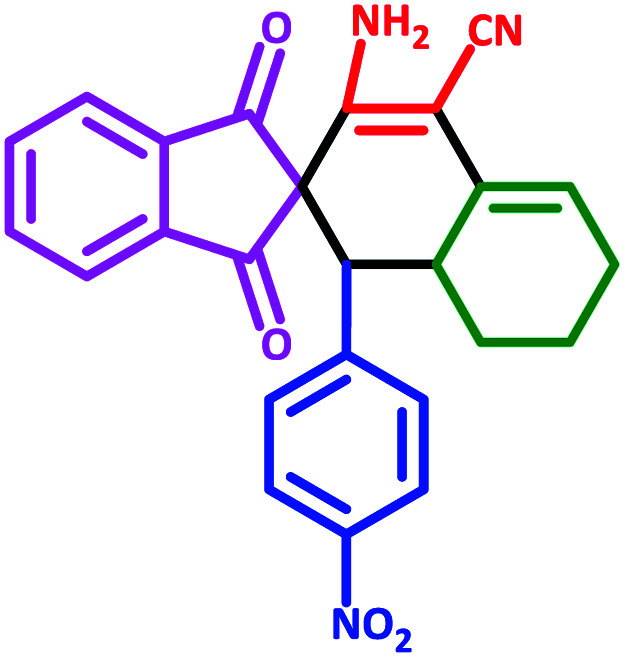	30	91	210–240 (Decompose)
2	5b	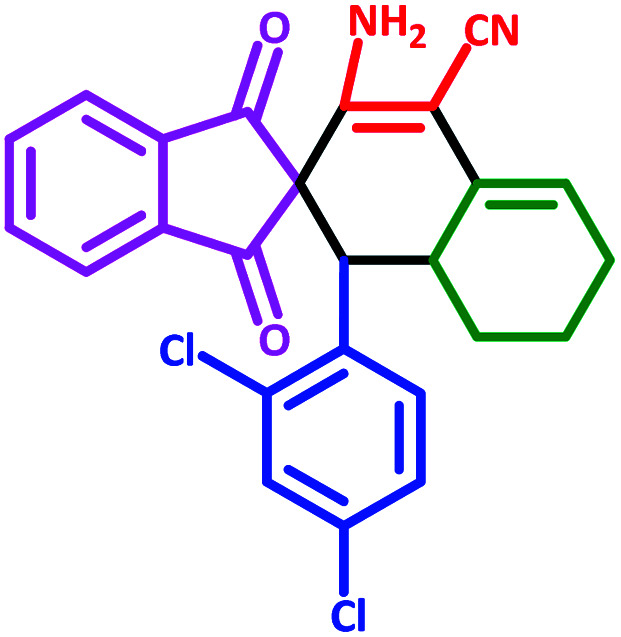	35	76	245–20 (Decompose)
3	5c	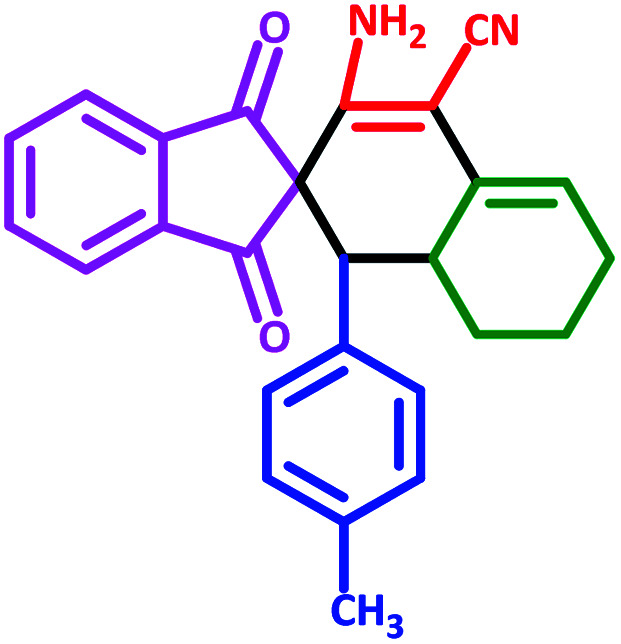	60	73	225–228 (Decompose)
4	5d	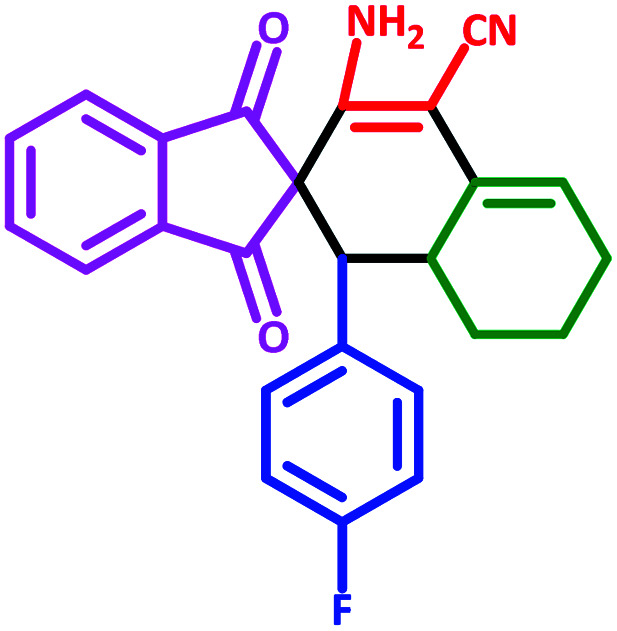	35	87	265–275 (Decompose)
5	5e	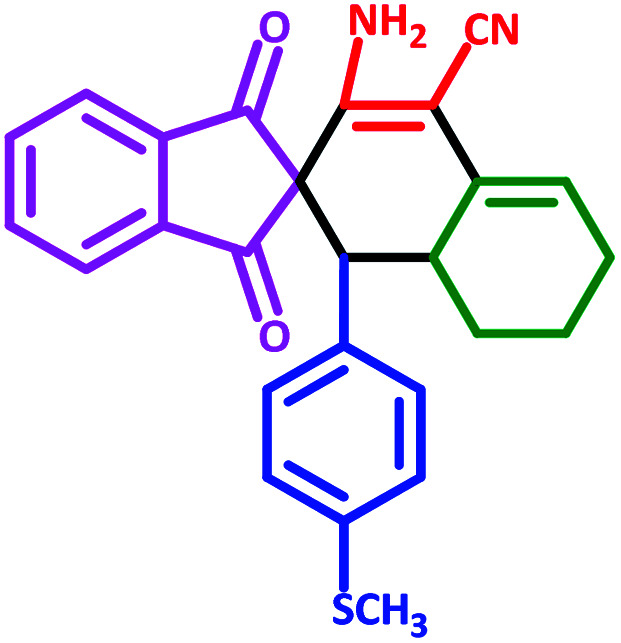	50	75	240–248 (Decompose)
6	5f	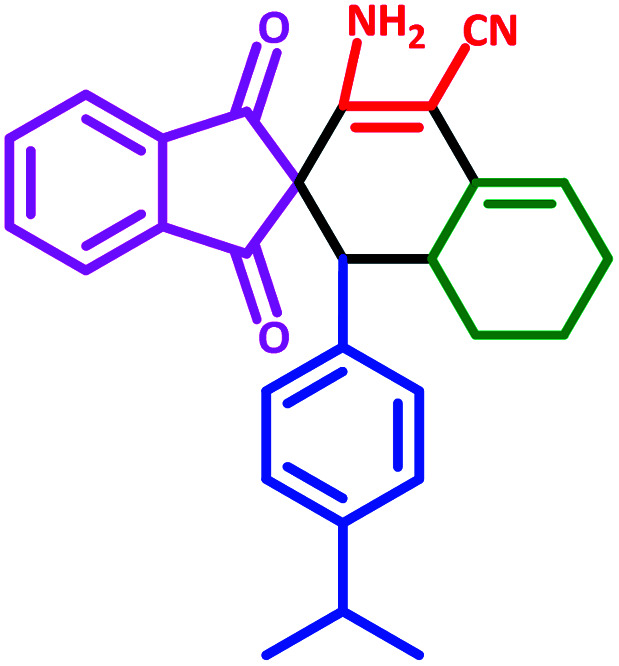	55	74	232–240 (Decompose)
7	5g	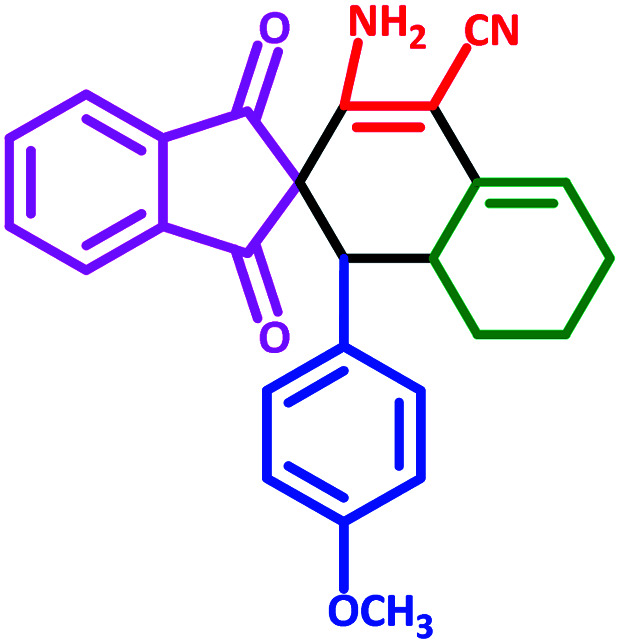	55	72	250–255 (Decompose)
8	5h	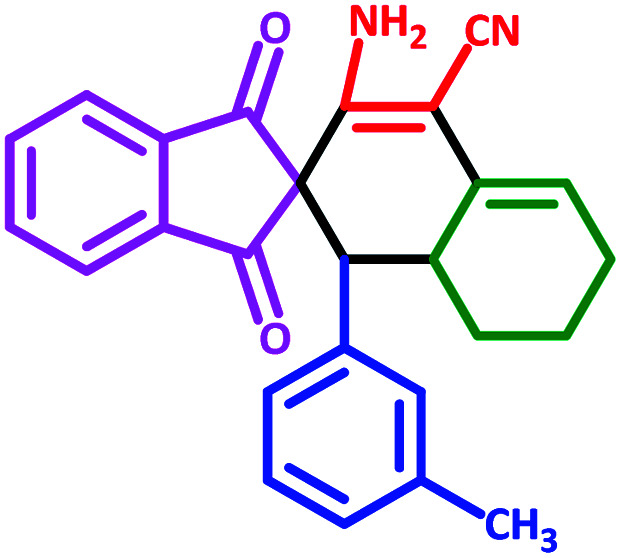	60	70	210–219 (Decompose)
9	5i	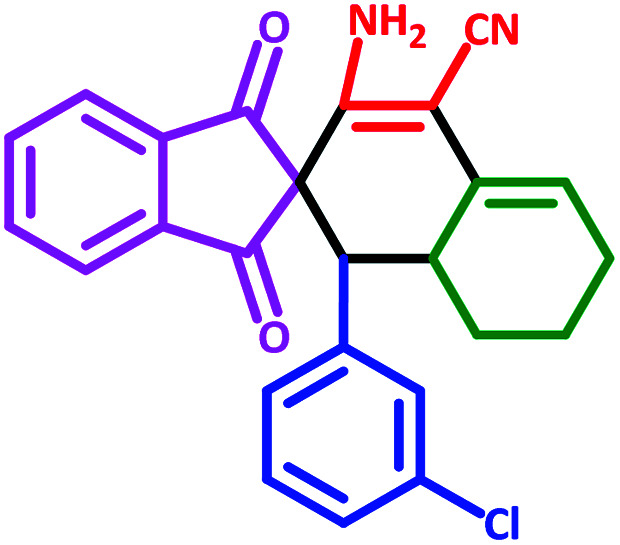	40	81	235–243 (Decompose)
10	5j	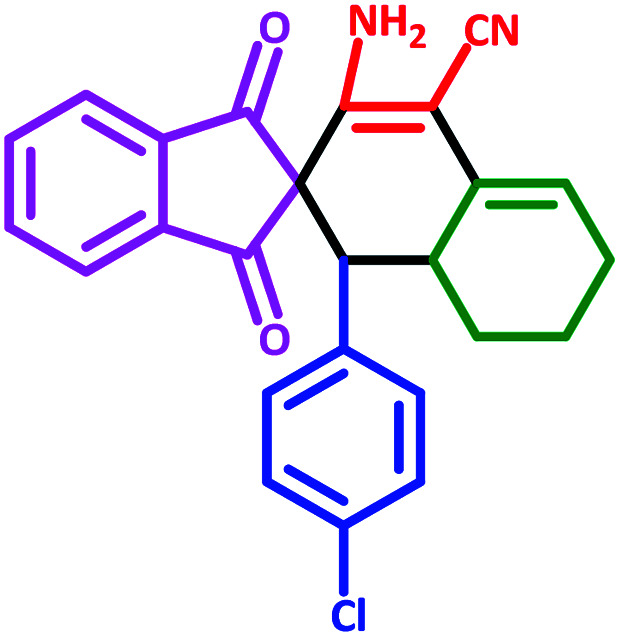	30	83	250–260 (Decompose)
11	5k	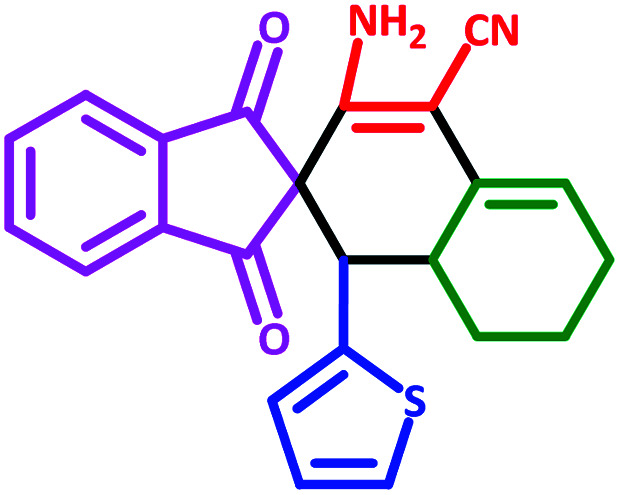	70	64	255–260 (Decompose)
12	5l	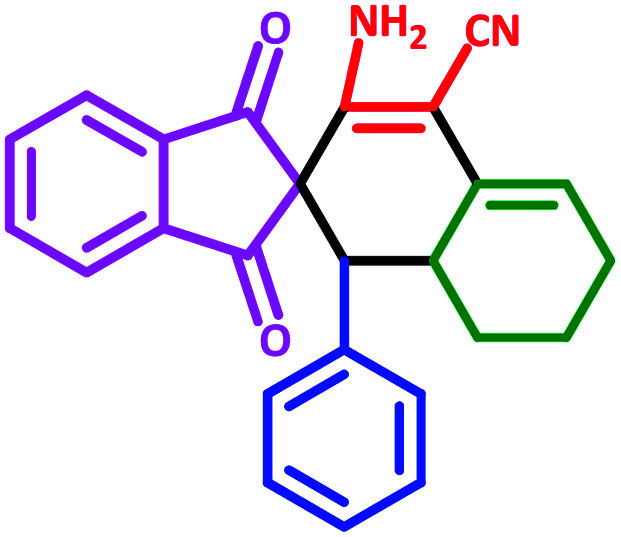	50	77	245–258 (Decompose)

a Reaction conditions: malononitrile (1 mmol), cyclohexanone (1 mmol), aromatic aldehydes (1 mmol), and 1,3-indandione (1 mmol) in presence Fe_3_O_4_@SiO_2_@L-tryptophan (20 mg) in EtOH under reflux conditions.

b Isolated yields.

### 2.3 Reusability of the Fe_3_O_4_@SiO_2_-l-tryptophan MNPs

**Fig. 8:**
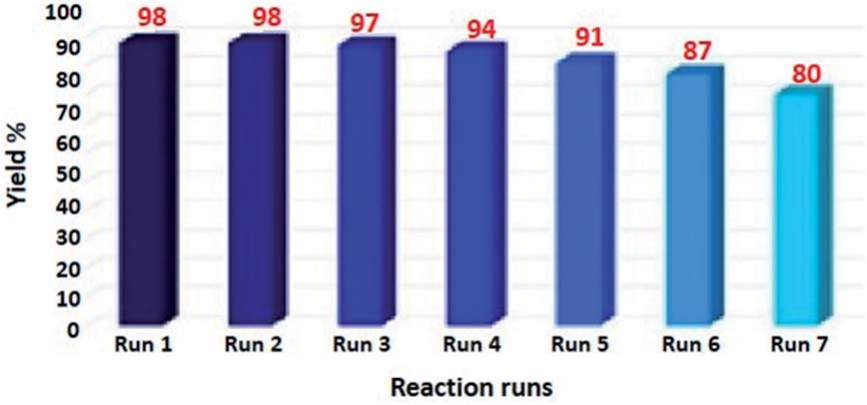
Recycling of Fe_3_O_4_@SiO_2_-l-tryptophan MNPs as the catalyst.

The reusability of the catalyst is well known as key characteristic properties. Herein, we have investigated the retrievability of Fe_3_O_4_@SiO_2_-l-tryptophan MNPs using the model reaction of malononitrile, cyclohexanone, 4-nitro benzaldehyde, and 1,3-indandione. As it can be viewed from Fig. 8, the catalytic activity of Fe_3_O_4_@SiO_2_-l-tryptophan declined from 98% in the fresh run to 80% after the completion of seven runs.

The prepared nanocatalyst was conveniently separated at the end of the reaction using a strong magnet, washed with EtOH and water, then dried at 60 °C, and reused seven times without excess purification.

The heterogeneous process was also checked for the model reaction. For this purpose, the catalyst was separated from the reaction mixture after 10 minutes and the reaction yield was estimated to be around 64%. Then, the reaction continued without the catalyst for another 20 minutes. The obtained product had no significant increase in the yield (∼67%). According to the above-mentioned results, the presence of the catalyst until at the end of the reaction is necessary to reach the best yield.

**Fig. 9:**
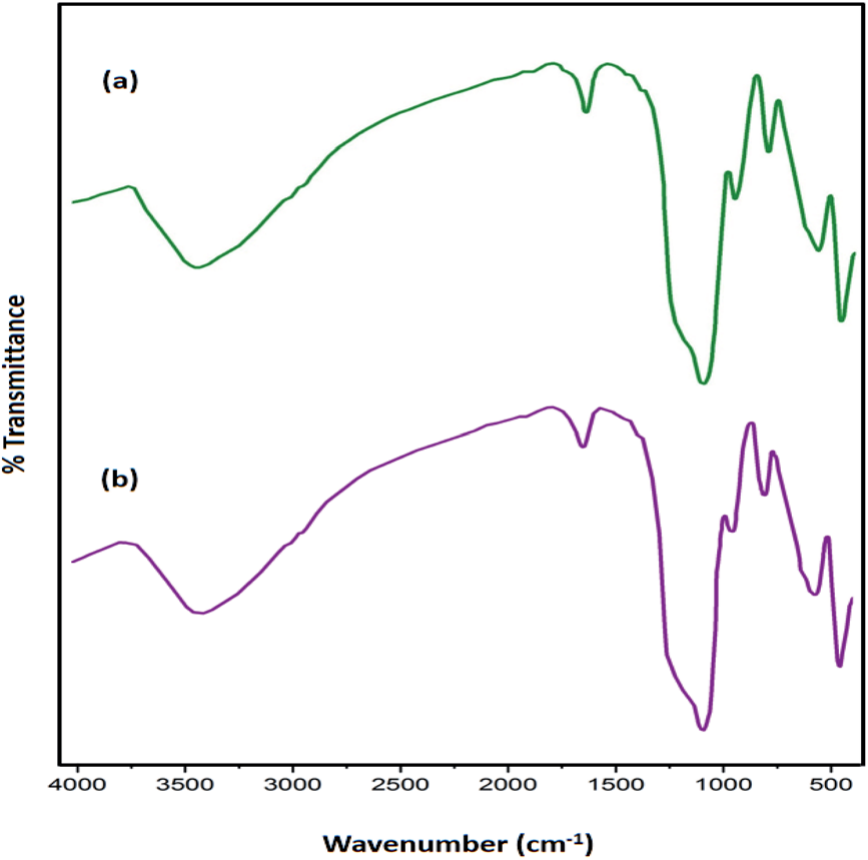
FT-IR spectra of Fe_3_O_4_@SiO_2_-l-tryptophan MNPs (a) before and (b) after seven reaction cycles.

The amount of the base in the catalyst before and after the cyclic test was quantitatively evaluated through ion-exchange pH analysis.^39^ Based on the acid–base titration measurement, the number of basic sites of the catalyst was approximately 1.250 mmol g^−1^ after being recycled for seven cycles, while the amount of basic sites in the fresh Fe_3_O_4_@SiO_2_-l-tryptophan nanocatalyst were estimated at 1.406 mmol g^−1^. Further, the chemical structure of the recovered catalyst after 7 cycles was confirmed using the FT-IR spectrum. As can be observed in Fig. 9, there is no considerable difference between FT-IR spectra of the fresh and reused magnetic nanocatalyst.

### 2.4 A plausible mechanism for the preparation of spiro[indene-2,2′-naphthalene]-4′-carbonitriles

**Scheme 3:**
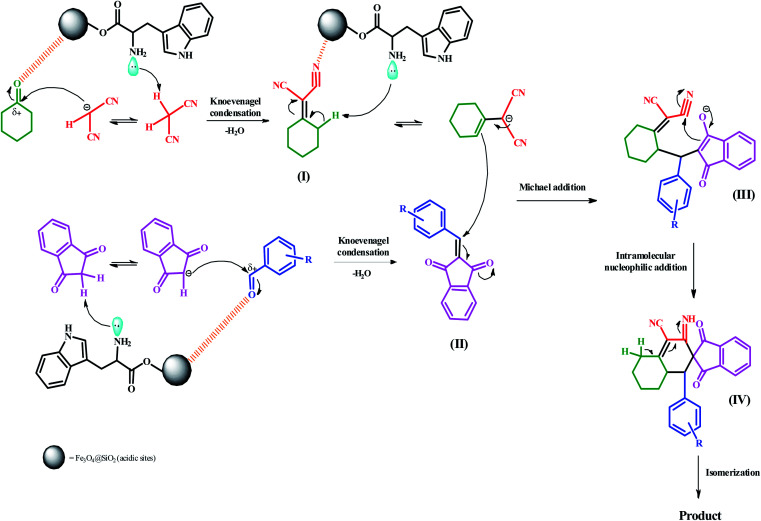
The probable mechanism for the preparation of spiro[indene-2,2′-naphthalene]-4′-carbonitrile compounds.

A feasible mechanism and catalytic cycle are illustrated in Scheme 3 for the preparation of spiro[indene-2,2′-naphthalene]-4′-carbonitriles. Initially, the reaction begins through acidic sites (electrostatic attraction) of Fe_3_O_4_@SiO_2_ nanocatalyst binds with the oxygen atom of the carbonyl group. Simultaneously, the acidic hydrogen of malononitrile is removed by lone pairs of the amino group of l-tryptophan. Afterward, the carbonyl group (C=O) of cyclohexanone is attacked by carbanion. The Knoevenagel condensation occurs to produce the intermediate (I). The same conditions have taken place between two other compounds. In other words, 1,3-indanedione and aromatic aldehydes underwent the Knoevenagel condensation and afforded intermediate (II). In continuing, basic sites of the nanocatalyst (amino group) removed the γ-proton of cyclohexylidene malononitrile (I) to afford the cyclohexylidene malononitrile carbanion. In the next step, the carbanion attacks the activated double bond of the intermediate (II) *via* the Michael addition to produce the intermediate (III). Then, intermediate (IV) is furnished by an intramolecular nucleophilic addition reaction. Ultimately, an isomerization results to form the final product.

## 3. Experimental

### 3.1 General

All solvents and reagents were purchased commercially and were utilized without any further purification. Fourier transform infrared (FT-IR) spectroscopy was performed using a Nicolet Magna-400 spectrometer (KBr pellets). ^1^H NMR was recorded in DMSO-d_6_ solvent using a Bruker DRX-400 spectrometer with tetramethylsilane (TMS) as the internal reference. XRD patterns were recorded using a Philips diffractometer and monochromatized Cu Kα radiation (*k* = 1.5406 Å). The morphological study of the nanoparticles was conducted using field emission scanning electron microscopy (FE-SEM) (model MIRA3). The electron dispersive X-ray (EDX) analysis of the catalyst was performed using an Oxford instrument company. Thermogravimetric analysis (TGA) was performed on a Mettler TA4000 system TG-50 at a heating rate of 10 K min^−1^ under N_2_ atmosphere. The magnetic properties of samples were measured using a magnetometer (VSM, PPMS-9T) at 300 K in Iran (Kashan University). Melting points were obtained using a Yanagimoto micromelting point apparatus and are uncorrected. The purity determination of the substrates and reaction monitoring were accomplished using TLC on silica-gel polygram SILG/UV 254 plates (from Merck Company).

### 3.2 General procedure for the synthesis of Fe_3_O_4_ nanoparticles

Fe_3_O_4_ nanoparticles were prepared using the chemical co-precipitation method according to our previous work with some modifications.^38,39^ Briefly, FeCl_3_·6H_2_O (16 mmol, 4.32 g) and FeCl_2_·4H_2_O (8 mmol, 1.6 g) were dissolved in 100 mL of deionized water under N_2_ protection. Then, the reaction temperature was increased to 80 °C and 25 mL NH_4_OH (25%) was added to the solution dropwise (pH = 12). After adding NH_4_OH to the solution, the color of the solution turned black. The reaction was stirred at 80 °C for 1 h under reflux conditions. The magnetic nanoparticles were separated from the solution using an external magnet and washed several times with deionized water and ethanol and then dried at 70 °C for 12 h in an oven.

### 3.3 Preparation of Fe_3_O_4_@SiO_2_

Fe_3_O_4_@SiO_2_ nanoparticles were obtained according to the reported method in the literature with some modifications.^40,41^ Magnetic nanoparticles (1 g) were dispersed in a solution of ethanol and water (40 : 10 mL) in an ultrasonic bath for 30 min. The pH was adjusted to 10 with an ammonia solution and 0.5 mL tetraethylorthosilicate (TEOS), which was added dropwise into the mixture over a period of 1 h. The resulting solution was stirred at 35–40 °C for 12 h. Fe_3_O_4_@SiO_2_ MNPs were separated from the solution by using an external magnet and washed with ethanol (3 × 15 mL) and dried at room temperature.

### 3.4 Preparation of Fe_3_O_4_@SiO_2_@l-tryptophan

Firstly, Fe_3_O_4_@SiO_2_ MNPs (1 g) were dispersed in dry ethanol (10 mL) using an ultrasonic bath for 30 min. Subsequently, H_2_SO_4_ (0.5 mL) and l-tryptophan (1.5 g) were added to the solution and heated under reflux conditions at 90 °C for 12 h. The resulting MNPs were collected by magnetic separation followed by washing with ethanol and water several times before being dried in an oven at 60 °C to give Fe_3_O_4_@SiO_2_@l-tryptophan as a light brown powder.

### 3.5 A common procedure for the synthesis of (5a–l)

**Scheme 4:**
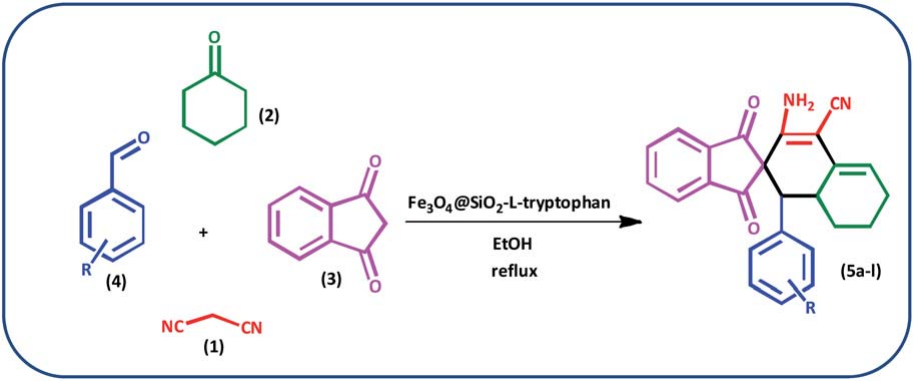
Preparation of spiro[indene-2,2′-naphthalene]-4′-carbonitrile derivatives by using Fe_3_O_4_@SiO_2_-l-tryptophan as the catalyst.

Typically, malononitrile (1) (1 mmol) and cyclohexanone (2) (1 mmol) were stirred in EtOH in the presence of Fe_3_O_4_@SiO_2_@l-tryptophan at room temperature (intermediate A). In the other test tube, 1,3-indanedione (3) (1 mmol) and various aromatic aldehydes (4) (1 mmol) were mixed and stirred under equal conditions (intermediate B). Then, intermediate A was added to intermediate B and the reaction was continued for the right time to achieve the desired product (Scheme 4). Upon completion of the time, the reaction was monitored by TLC (*n*-hexane/ethyl acetate: 6 : 4) the crude precipitate was filtered off and purified by recrystallization from ethanol to obtain the pure product. All products were characterized by melting point (m.p.), FT-IR, ^1^H NMR, ^13^C NMR and elemental microanalysis.

#### 3.5.1 3′-Amino-1′-(4-nitrophenyl)-1,3-dioxo-1,3,6′,7′,8′,8*a*′-hexahydro-1′*H*-spiro[indene-2,2′-naphthalene]-4′-carbonitrile (5a)

Yellow solid: m.p.: 210–240 (Decompose). IR (cm^−1^): 3431, 3353, 3254, 2941, 2849, 2201, 1700, 1645, 1590, 1349, 1238. ^1^H NMR (400 MHz, DMSO-d_6_) *δ* (ppm): 7.87–7.83 (m, 2H), 7.76–7.74 (m, 3H), 7.67 (d, 1H, *J* = 7.6 Hz), 7.16 (d, 1H, *J* = 9.2 Hz), 7.07 (d, 1H, *J* = 8 Hz), 6.27 (s, 2H, NH_2_, D_2_O exchangeable), 5.64 (s, 1H, vinylic), 3.26 (d, 1H, *J* = 12.8 Hz, –CH), 3.14 (m, 1H, CH), 2.22–2.17 (m, 1H, CH_2_), 2.07–2.04 (m, 1H, CH_2_), 1.64 (m, 1H, CH_2_), 1.43 (m, 1H, CH_2_), 1.28–1.25 (m, 1H, CH_2_), 0.82 (q, 1H, *J* = 12.4 Hz, CH_2_). Anal. calcd for C_25_H_19_N_3_O_4_: C, 70.58; H, 4.50; N, 9.88; O, 15.04; found: C, 70.55; H, 4.47; N, 9.89; O, 15.03.

#### 3.5.2 3′-Amino-1′-(2,4-dichlorophenyl)-1,3-dioxo-1,3,6′,7′,8′,8*a*′-hexahydro-1′*H*-spiro[indene-2,2′-naphthalene]-4′-carbonitrile (5b)

Yellow solid: m.p.: 245–250 (Decompose). IR (cm^−1^): 3459, 3366, 3084, 2923, 2864, 2208, 1703, 1632, 1588, 1241. ^1^H NMR (400 MHz, DMSO-d_6_) *δ* (ppm): 7.84–7.78 (m, 3H), 7.74 (d, 1H, *J* = 6.8 Hz), 7.35 (d, 1H, *J* = 1.6 Hz), 7.07 (dd, 1H, *J* = 1.6 Hz, *J* = 8.4 Hz), 6.96 (d, 1H, *J* = 8.4 Hz), 6.29 (s, 2H, NH_2_, D_2_O exchangeable), 5.63 (s, 1H, vinylic), 3.72 (d, 1H, *J* = 12.4 Hz, –CH), 2.97 (m, 1H, –CH), 2.19–2.07 (m, 2H, CH_2_), 1.62 (m, 1H, CH_2_), 1.36 (m, 1H, CH_2_), 1.17–1.14, (m, 1H, CH_2_), 0.70 (q, 1H, *J* = 12.8 Hz, CH_2_). Anal. calcd for C_25_H_18_Cl_2_N_2_O_2_: C, 66.83; H, 4.04; Cl, 15.78; N, 6.23; O, 7.12; found: C, 66.82; H, 4.02; Cl, 15.77; N, 6.20; O, 7.10.

#### 3.5.3 3′-Amino-1,3-dioxo-1′-(*p*-tolyl)-1,3,6′,7′,8′,8*a*′-hexahydro-1′*H*-spiro[indene-2,2′-naphthalene]-4′-carbonitrile (5c)

Yellow solid: m.p: 225–228 (Decompose). IR (cm^−1^): 3409, 3345, 3244, 3020, 2922, 2201, 1704, 1657, 1587, 1243. ^1^H NMR (400 MHz, DMSO-d_6_) *δ* (ppm): 7.75 (m, 3H), 7.66 (d, 1H, *J* = 7.2 Hz), 6.77–6.73 (m, 3H), 6.60 (d, 1H, *J* = 8 Hz), 6.15 (s, 2H, NH_2_, D_2_O exchangeable), 5.59 (s, 1H, vinylic), 3.07 (m, 1H, –CH), 2.97 (d, 1H, *J* = 12.4 Hz, –CH), 2.19–2.15 (m, 2H, CH_2_), 1.99 (s, 3H, –CH_3_), 1.63 (m, 1H, CH_2_), 1.38–1.31 (m, 2H, CH_2_), 0.71 (q, 1H, *J* = 13.2 Hz, CH_2_). Anal. calcd for C_26_H_22_N_2_O_2_: C, 79.16; H, 5.62; N, 7.10; O, 8.11; found: C, 79.17; H, 5.63; N, 7.11; O, 8.09.

#### 3.5.4 3′-Amino-1′-(4-fluorophenyl)-1,3-dioxo-1,3,6′,7′,8′,8*a*′-hexahydro-1′*H*-spiro[indene-2,2′-naphthalene]-4′-carbonitrile (5d)

Yellow solid: m.p.: 265–275 (Decompose). IR (cm^−1^): 3448, 3361, 2911, 2198, 1736, 1695, 1591, 1234. ^1^H NMR (400 MHz, DMSO-d_6_) *δ* (ppm): 7.76 (d, 3H, *J* = 8.4 Hz), 7.67 (d, 1H, *J* = 6.4 Hz), 6.87–6.86 (m, 1H), 6.79 (d, 3H, *J* = 8.4 Hz), 6.20 (s, 2H, NH_2_, D_2_O exchangeable), 5.61 (s, 1H, vinylic), 3.07 (m, 2H, 2CH), 2.20–2.16 (m, 1H, CH_2_), 2.07–2.04 (m, 1H, CH_2_), 1.65 (m, 1H, CH_2_), 1.40 (m, 1H, CH_2_), 1.34–1.31 (m, 1H, CH_2_), 0.72 (q, 1H, *J* = 11.6 Hz, CH_2_). ^13^C NMR (100 MHz, DMSO-d_6_) *δ* (ppm): 199.60, 199.18, 166.33, 159.78, 151.25, 142.87, 142.08, 136.22, 136.11, 132.89, 132.81, 131.93, 131.15, 128.36, 123.28, 122.70, 117.01, 115.00, 80.98, 63.15, 51.20, 32.99, 27.31, 24.93, 21.56. Anal. calcd for C_25_H_19_FN_2_O_2_: C, 75.36; H, 4.81; F, 4.77; N, 7.03; O, 8.03; found: C, 75.36; H, 4.80; F, 4.75; N, 7.04; O, 8.01.

#### 3.5.5 3′-Amino-1′-(4-(methylthio)phenyl)-1,3-dioxo-1,3,6′,7′,8′,8*a*′-hexahydro-1′*H*-spiro[indene-2,2′-naphthalene]-4′-carbonitrile (5e)

Yellow solid: m.p.: 240–248 (Decompose). IR (cm^−1^): 3422, 3339, 2928, 2202, 1703, 1635, 1589, 1242. ^1^H NMR (400 MHz, DMSO-d_6_) *δ* (ppm): 7.76 (m, 3H), 7.69 (m, 1H), 6.87–6.83 (m, 2H), 6.77 (d, 1H, *J* = 7.6 Hz), 6.67 (d, 1H, *J* = 7.6 Hz), 6.17 (s, 2H, NH_2_, D_2_O exchangeable), 5.60 (s, 1H, vinylic), 3.05 (m, 1H, –CH), 2.99 (d, 1H, *J* = 12.8 Hz, –CH), 2.24 (s, 3H, –SCH_3_), 2.15 (m, 1H, CH_2_), 2.07 (m, 1H, CH_2_), 1.64 (m, 1H, CH_2_), 1.39–1.30 (m, 2H, CH_2_), 0.72 (q, 1H, *J* = 11.6 Hz, CH_2_). ^13^C NMR (100 MHz, DMSO-d_6_) *δ* (ppm): 200.13, 199.68, 151.84, 143.37, 142.66, 137.65, 136.67, 136.54, 132.75, 132.02, 131.76, 127.33, 126.28, 125.87, 123.83, 123.22, 118.08, 117.47, 82.93, 63.61, 52.01, 33.52, 27.88, 25.44, 22.08, 15.01. Anal. calcd for C_26_H_22_N_2_O_2_S: C, 73.21; H, 5.20; N, 6.57; O, 7.50; S, 7.52; found: C, 73.22; H, 5.25; N, 6.51; O, 7.47; S, 7.51.

#### 3.5.6 3′-Amino-1′-(4-isopropylphenyl)-1,3-dioxo-1,3,6′,7′,8′,8*a*′-hexahydro-1′*H*-spiro[indene-2,2′-naphthalene]-4′-carbonitrile (5f)

Yellow solid: m.p: 232–240 (Decompose). IR (cm^−1^): 3432, 3343, 3248, 2954, 2197, 1702, 1648, 1586, 1241. ^1^H NMR (400 MHz, DMSO-d_6_) *δ* (ppm): 7.70–7.69 (m, 3H), 7.63 (m, 1H), 6.79–6.76 (m, 3H), 6.61 (d, 1H, *J* = 7.6 Hz), 6.18 (s, 2H, NH_2_, D_2_O exchangeable), 5.61 (s, 1H, vinylic), 3.08 (m, 1H, CH), 2.99 (d, 1H, *J* = 12.8 Hz, CH), 2.57 (m, 1H, CH(CH_3_)_2_), 2.21–2.17 (m, 1H, CH_2_), 2.09 (m, 1H, CH_2_), 1.67 (m, 1H, CH_2_), 1.44–1.42 (m, 2H, CH_2_), 0.90 (d, 6H, 2CH_3_), 0.75 (q, 1H, *J* = 11.6 Hz, CH_2_). Anal. calcd for C_28_H_26_N_2_O_2_: C, 79.59; H, 6.20; N, 6.63; O, 7.57; found: C, 79.57; H, 6.22; N, 6.58; O, 7.56.

#### 3.5.7 3′-Amino-1′-(4-methoxyphenyl)-1,3-dioxo-1,3,6′,7′,8′,8*a*′-hexahydro-1′*H*-spiro[indene-2,2′-naphthalene]-4′-carbonitrile (5g)

Yellow solid: m.p.: 250–255 (Decompose). IR (cm^−1^): 3424, 3341, 3245, 2920, 2199, 1701, 1654, 1510, 1242. ^1^H NMR (400 MHz, DMSO-d_6_) *δ* (ppm): 7.75–7.74 (m, 2H), 7.69–7.66 (m, 2H), 6.74 (d, 1H, *J* = 8.8 Hz), 6.64 (d, 1H, *J* = 8.4 Hz), 6.54–6.49 (m, 2H), 6.14 (s, 2H, NH_2_, D_2_O exchangeable), 5.59 (s, 1H, vinylic), 3.50 (s, 3H, –OCH_3_), 3.02 (m, 1H, CH), 2.97 (d, 1H, *J* = 12.4 Hz, CH), 2.20–2.15 (m, 1H, CH_2_), 2.07 (m, 1H, CH_2_), 1.63 (m, 1H, CH_2_), 1.34–1.28 (m, 2H, CH_2_), 0.71 (q, 1H, *J* = 12.8 Hz, CH_2_). Anal. calcd for C_26_H_22_N_2_O_3_: C, 76.08; H, 5.40; N, 6.82; O, 11.69; found: C, 76.06; H, 5.41; N, 6.80; O, 11.67.

#### 3.5.8 3′-Amino-1,3-dioxo-1′-(*m*-tolyl)-1,3,6′,7′,8′,8*a*′-hexahydro-1′*H*-spiro[indene-2,2′-naphthalene]-4′-carbonitrile (5h)

Yellow solid: m.p.: 210–219 (Decompose). IR (cm^−1^): 3442, 3345, 2915, 2196, 1700, 1636, 1593, 1240. ^1^H NMR (400 MHz, DMSO-d_6_) *δ* (ppm): 7.68–7.60 (m, 4H), 6.86–6.80 (m, 1H), 6.69–6.64 (m, 1H), 6.62–6.60 (m, 1H), 6.51–6.49 (m, 1H), 6.18 (d, 2H, NH_2_, D_2_O exchangeable), 5.64 (s, 1H, vinylic), 3.05 (m, 1H, CH), 2.98 (d, 1H, *J* = 11.6 Hz, CH), 2.20 (m, 1H, CH_2_), 2.15 (m, 1H, CH_2_), 2.04 (d, 3H, –CH_3_), 1.64 (m, 1H, CH_2_), 1.39–1.35 (m, 2H, CH_2_), 0.73 (q, 1H, *J* = 12.4 Hz, CH_2_). ^13^C NMR (100 MHz, DMSO-d_6_) *δ* (ppm): 200.16, 199.54, 152.01, 143.47, 142.89, 137.58, 136.47, 136.28, 132.05, 132.13, 131.83, 128.74, 128.37, 127.89, 123.49, 122.83, 118.15, 117.40, 82.89, 63.92, 52.84, 33.43, 27.95, 25.46, 21.30, 18.21. Anal. calcd for C_26_H_22_N_2_O_2_: C, 79.16; H, 5.62; N, 7.10; O, 8.11; found: C, 79.18; H, 5.63; N, 7.08; O, 8.10.

#### 3.5.9 3′-Amino-1′-(3-chlorophenyl)-1,3-dioxo-1,3,6′,7′,8′,8*a*′-hexahydro-1′*H*-spiro[indene-2,2′-naphthalene]-4′-carbonitrile (5i)

Yellow solid: m.p.: 235–243 (Decompose). IR (cm^−1^): 3447, 3354, 3245, 2913, 2198, 1696, 1635, 1585, 1239. ^1^H NMR (400 MHz, DMSO-d_6_) *δ* (ppm): 7.81–7.72 (m, 4H), 6.98 (m, 1H), 6.80 (m, 1H), 6.72 (d, 2H, *J* = 5.2 Hz), 6.23 (d, 2H, NH_2_, D_2_O exchangeable), 5.61 (s, 1H, vinylic), 3.10 (m, 1H, CH), 3.05 (d, 1H, *J* = 12 Hz, CH), 2.20–2.16 (m, 1H, CH_2_), 2.06 (m, 1H, CH_2_), 1.65 (m, 1H, CH_2_), 1.43 (m, 1H, CH_2_), 1.34–1.31 (m, 1H, CH_2_), 0.74 (q, 1H, *J* = 11.6 Hz, CH_2_). ^13^C NMR (100 MHz, DMSO-d_6_) *δ* (ppm): 200.79, 199.34, 151.59, 143.31, 142.54, 138.72, 136.68, 133.51, 131.07, 130.46, 127.70, 126.70, 125.69, 123.80, 123.24, 122.91, 118.03, 117.64, 82.92, 63.54, 51.95, 33.31, 27.72, 25.41, 21.98. Anal. calcd for C_25_H_19_ClN_2_O_2_: C, 72.37; H, 4.62; Cl, 8.55; N, 6.75; O, 7.71; found: C, 72.34; H, 4.63; Cl, 8.54; N, 6.76; O, 7.72.

#### 3.5.10 3′-Amino-1′-(4-chlorophenyl)-1,3-dioxo-1,3,6′,7′,8′,8*a*′-hexahydro-1′*H*-spiro[indene-2,2′-naphthalene]-4′-carbonitrile (5j)

Yellow solid: m.p.: 250–260 (Decompose). IR (cm^−1^): 3449, 3357, 3247, 2913, 2199, 1737, 1696, 1635, 1588, 1241. ^1^H NMR (400 MHz, DMSO-d_6_) *δ* (ppm): 7.77 (d, 3H, *J* = 9.6 Hz), 7.69 (d, 1H, *J* = 7.2 Hz), 7.04 (d, 2H, *J* = 8.4 Hz), 6.86 (d, 1H, *J* = 8 Hz), 6.76 (d, 1H, *J* = 7.2 Hz), 6.22 (s, 2H, NH_2_, D_2_O exchangeable), 5.61 (s, 1H, vinylic), 3.06 (m, 2H, 2CH), 2.20–2.15 (m, 1H, CH_2_), 2.07 (m, 1H, CH_2_), 1.65 (m, 1H, CH_2_), 1.42–1.40 (m, 1H, CH_2_), 1.32–1.29 (m, 1H, CH_2_), 0.73 (q, 1H, *J* = 11.6 Hz, CH_2_). Anal. calcd for C_25_H_19_ClN_2_O_2_: C, 72.37; H, 4.62; Cl, 8.55; N, 6.75; O, 7.71; found: C, 72.30; H, 4.65; Cl, 8.54; N, 6.73; O, 7.72.

#### 3.5.11 3′-Amino-1,3-dioxo-1′-(thiophen-2-yl)-1,3,6′,7′,8′,8*a*′-hexahydro-1′*H*-spiro[indene-2,2′-naphthalene]-4′-carbonitrile (5k)

Yellow solid: m.p.: 255–260 (Decompose). IR (cm^−1^): 3406, 3346, 3245, 2917, 2201, 1703, 1657, 1586, 1245. ^1^H NMR (400 MHz, DMSO-d_6_) *δ* (ppm): 7.82–7.77 (m, 4H), 7.09 (s, 1H), 6.61 (s, 1H), 6.54 (s, 1H), 6.18 (s, 2H, NH_2_, D_2_O exchangeable), 5.60 (s, 1H, vinylic), 3.45 (d, 1H, *J* = 12 Hz, CH), 2.96 (m, 1H, CH), 2.21–2.16 (m, 1H, CH_2_), 2.09 (m, 1H, CH_2_), 1.69 (m, 1H, CH_2_), 1.46 (m, 2H, CH_2_), 0.84 (q, 1H, *J* = 12 Hz, CH_2_). Anal. calcd for C_23_H_18_N_2_O_2_S: C, 71.48; H, 4.69; N, 7.25; O, 8.28; S, 8.30; found: C, 71.47; H, 4.69; N, 7.27; O, 8.25; S, 8.27.

#### 3.5.12 3′-Amino-1,3-dioxo-1′-phenyl-1,3,6′,7′,8′,8*a*′-hexahydro-1′*H*-spiro[indene-2,2′-naphthalene]-4′-carbonitrile (5l)

Yellow solid: m.p.: 245–258 (Decompose). IR (cm^−1^): 3451, 3363, 2910, 2199, 1696, 1635, 1590, 1242. ^1^H NMR (400 MHz, DMSO-d_6_) *δ* (ppm): 7.73 (s, 3H), 7.65–7.64 (m, 1H), 6.95–6.90 (m, 3H), 6.85 (d, 1H, *J* = 8.4 Hz), 6.72 (d, 1H, *J* = 7.6 Hz), 6.18 (s, 2H, NH_2_, D_2_O exchangeable), 5.60 (s, 1H, vinylic), 3.09–3.07 (m, 1H, CH), 3.02 (d, 1H, *J* = 12 Hz, CH), 2.21–2.16 (m, 1H, CH_2_), 2.08 (m, 1H, CH_2_), 1.64 (m, 1H, CH_2_), 1.41–1.33 (m, 2H, CH_2_), 0.73 (q, 1H, *J* = 13.2 Hz, CH_2_). ^13^C NMR (100 MHz, DMSO-d_6_) *δ* (ppm): 199.63, 199.21, 166.44, 151.41, 142.88, 142.17, 136.07, 135.93, 135.68, 131.00, 128.53, 128.08, 127.32, 126.31, 123.22, 122.64, 117.64, 116.91, 80.99, 63.19, 52.07, 32.92, 27.40, 24.95, 21.59. Anal. calcd for C_25_H_20_N_2_O_2_: C, 78.93; H, 5.30; N, 7.36; O, 8.41; found: C, 78.91; H, 5.29; N, 7.37; O, 8.40.

## 4. Conclusion

In conclusion, Fe_3_O_4_@SiO_2_-l-tryptophan magnetic nanocatalyst as green, magnetically recyclable, and environmentally friendly, was prepared and fully characterized. We applied this catalyst for the synthesis of spiro[indene-2,2′-naphthalene]-4′-carbonitrile derivatives *via* a four-component reaction of malononitrile, cyclohexanone, aromatic aldehydes and 1,3-indandione. Excellent yields in short reaction time, facile, soft reaction conditions, and high atom economy are some of the advantages of this procedure.

## Conflicts of interest

There are no conflicts to declare.

## Supplementary Material

## References

[cit1] Younus H. A., Al-Rashida M., Hameed A., Uroos M., Salar U., Rana S., Khan K. M. (2021). Expert Opin. Ther. Pat..

[cit2] Ruijter E., Orru R. V. A. (2013). Drug Discovery Today: Technol..

[cit3] Luo J., Chen G. S., Chen S. J., Li Z. D., Liu Y. L. (2021). Chem.–Eur. J..

[cit4] Volla C. M. R., Atodiresei I., Rueping M. (2014). Chem. Rev..

[cit5] Komogortsev A. N., Melekhina V. G., Lichitsky B. V., Minyaev M. E. (2020). Tetrahedron Lett..

[cit6] Chaudhary A. (2021). Mol. Diversity.

[cit7] Biesen L., Muller J. J. (2020). Adv. Synth. Catal..

[cit8] Dhokne P., Sakla A. P., Shankaraiah N. (2021). Eur. J. Med. Chem..

[cit9] Sampath S., Vadivelu M., Ravindran R., Perumal P. T., Velkannan V., Karthikeyan K. (2020). ChemistrySelect.

[cit10] Toumi A., Boudriga S., Hamden K., Sobeh M., Cheurfa M., Askri M., Knorr M., Strohmann C., Brieger L. (2020). Bioorg. Chem..

[cit11] Yan C., Sun J., Yan C. G. (2021). Chin. Chem. Lett..

[cit12] Kaur B. P., Kaur J., Singh Chimni S. (2021). RSC Adv..

[cit13] Mani K. S., Kaminsky W., Rajendran S. P. (2018). New J. Chem..

[cit14] Zhan S. C., Fang R. J., Sun J., Yan C. G. (2021). J. Org. Chem..

[cit15] Sankara C. S., Namboothiri I. N. N. (2021). Org. Lett..

[cit16] Kaur N., Singh P., Banerjee P. (2021). Adv. Synth. Catal..

[cit17] Nagaraju S., Sathish K., Satyanarayana N., Paplal B., Kashinath D. (2020). J. Heterocycl. Chem..

[cit18] Sudhapriya N., Perumal P. T., Balachandran C., Ignacimuthu S., Sangeetha M., Doble M. (2014). Eur. J. Med. Chem..

[cit19] Khanna G., Saluja P., Khurana J. M. (2016). Tetrahedron Lett..

[cit20] Lohar T., Kumbhar A., Patil A., Kamat S., Salunkhe R. (2019). Res. Chem. Intermed..

[cit21] Fatimah I., Fadillah G., Yudha S. P. (2021). Arabian J. Chem..

[cit22] Li X., Li W., Wang M., Liao Z. (2021). J. Controlled Release.

[cit23] Sun C., Lee J. S. H., Zhang M. (2008). Adv. Drug Delivery Rev..

[cit24] Baig R. B. N., Varma R. S. (2013). Chem. Commun..

[cit25] Yallapu M. M., Othman S. F., Curtis E. T., Gupta B. K., Jaggi M., Chauhan S. C. (2011). Biomaterials.

[cit26] Liu S., Yu B., Wang S., Shen Y., Cong H. (2020). Adv. Colloid Interface Sci..

[cit27] Lavorato G. C., Das R., Masa J. A., Phan M. H., Srikanth H. (2021). RSC Adv..

[cit28] Deng J., Mo L. P., Zhao F. Y., Zhang Z. H., Liu S. X. (2012). ACS Comb. Sci..

[cit29] Sepehrmnsourie H., Zarei M., Zolfigol M. A., Babaee S., Rostamnia S. (2021). Sci. Rep..

[cit30] Liu S., Yu B., Wang S., Shen Y., Cong H. (2020). Adv. Colloid Interface Sci..

[cit31] Jiang Z., Yang H., Han X., Luo J., Wong M. W., Lu Y. (2010). Org. Biomol. Chem..

[cit32] Luo S., Mi X., Zhang L., Liu S., Xu H., Cheng J.-P. (2006). Angew. Chem., Int. Ed..

[cit33] Gruttadauria M., Giacalone F., Marculescu A. M., Notoa R. (2008). Adv. Synth. Catal..

[cit34] Zamboulis A., Rahier N. J., Gehringer M., Cattoën X., Niel G., Bied C., Moreau J. J. E., Man M. W. C. (2009). Tetrahedron: Asymmetry.

[cit35] Ghorbani-Choghamarani A., Azadi G. (2016). Appl. Organomet. Chem..

[cit36] Gonzalez-Domínguez J. M., Gonzalez M., Anson-Casaos A., Díez-Pascual A. M., Gomez M. A., Martínez M. T. (2011). J. Phys. Chem. C.

[cit37] Solvents and Solvent Effects in Organic Chemistry, C. Reichardt, WILEY-VCH Verlag GmbH & Co. KGaA, copyright 8, third edn, 2003, Weinheim, ISBN: 3-527-30618-8

[cit38] Alemi-Tameh F., Safaei-Ghom J., Mahmoudi-Hashemi M., Shahbazi-Alavi H. (2016). RSC Adv..

[cit39] Oboudatian H. S., Naeimi H., Moradian M. (2021). RSC Adv..

[cit40] Safaei-Ghom J., Masoomi R., Hamadanian M., Naseh S. (2016). New J. Chem..

[cit41] Eidi E., Kassaee M. Z., Nasresfahani Z., Cummings P. T. (2018). Appl. Organomet. Chem..

